# Second victims of obstetric care – Support for healthcare professionals in KwaZulu-Natal, South Africa

**DOI:** 10.4102/sajpsychiatry.v28i0.1663

**Published:** 2022-02-24

**Authors:** Puvashnee Nydoo, Basil J. Pillay, Thajasvarie Naicker, Jagidesa Moodley

**Affiliations:** 1 Department of Obstetrics and Gynaecology, College of Health Sciences, University of KwaZulu-Natal, Durban, South Africa; 2 Department of Behavioural Medicine, College of Health Sciences, University of KwaZulu-Natal, Durban, South Africa; 3 Department of Optics and Imaging Centre, College of Health Sciences, University of KwaZulu-Natal, Durban, South Africa; 4 Department of Women’s Health and HIV Research Group, College of Health Sciences, University of KwaZulu-Natal, Durban, South Africa

## Background

Research on supportive interventions for healthcare professionals involved in adverse obstetric events in South Africa (SA) is limited. Involvement in adverse obstetric events has the potential to affect the personal and professional lives of healthcare professionals.^1^ Psychological distress and negative emotional reactions, with symptoms associated with posttraumatic stress disorder (PTSD), have been reported.^2^ Some individuals’ responses are so severe that they never fully recover, causing them to leave the profession altogether and even to consider or commit suicide.^3^ Support is crucial to prevent healthcare professionals from becoming second victims, to improve their personal and professional lives and to prevent subsequent negative impact to themselves and to their management of current and future patients.^2^ Protocols for the management of patients of adverse events are available within healthcare systems in most countries including SA. However, management and support processes for healthcare professionals are less clear or non-existent.^4,5,6^ Timely and effective emotional support can alleviate a healthcare professional’s second victim response.^7^ The provision of support by an organisation, accompanied by a tolerant and non-punitive working environment, can dramatically reduce the intensity of the second-victim response.^2,5^

## Aim

This study was conducted to ascertain the support-seeking behaviour of healthcare professionals involved in adverse obstetric events and to explore the organisational support in public sector hospitals in KwaZulu-Natal (KZN), SA.

## Method

A cross-sectional survey-based analysis of 231 obstetric healthcare professionals (midwives: *n* = 100; medical interns: *n* = 49; medical officers: *n* = 37; registrars: *n* - 20; specialists: *n* = 25) across nine public sector hospitals in KZN was conducted from January 2018 to August 2019. A self-report ‘Adverse Event Check List’ was developed for this study. Socio-demographic factors and context-specific questions relating to the adverse events were included in the Adverse Event Check List. Two standard validated rating scales were utilised as measures of psychological distress (Impact of Event Scale-Revised [IES-R])^8^ and shame and self-esteem (Internalized Shame Scale [ISS]).^9^ The IES-R had a scoring range of 0–88.^8^ For the purpose of this study, we used cut-off scores to determine four psychological distress severity symptom groups: no distress (≤ 23), clinical concern for PTSD (24–32), probable diagnosis of PTSD (33–36) and severe psychological distress (≥ 37). The ISS had a scoring range of 0–96.^9^ Cut-off scores for internalised shame were as follows: 0–49 (no display of internalised shame), 50–59 (painful, possibly problematic levels of internalised shame) and 60–96 (extreme levels of shame and likely to be associated with more severe symptoms of depression and/or anxiety). The ISS also allowed us to measure an individual’s self-esteem. The self-esteem cut-offs used were: 0–17 (direction of low self-esteem) and 18–24 (indicative of positive self-esteem). The questionnaires were pilot-tested on a small group of medical professionals before the commencement of the study.

## Ethical considerations

Ethical approval was obtained from the University of KwaZulu-Natal Biomedical Research Ethics Committee, reference number: BE484/17.

## Results

### Patient socio-demographic data

The age of the participants ranged from 22 to 66 (*M* ± 36.2; standard deviation [SD]: 9.7) years. Regarding health professional’s basic qualifications, 100 (43.3%), were midwives, 49 (21.2%) were medical interns, 25 (10.8%) were specialist obstetricians, 37 (16%) were medical officers and 20 (8.7%) were registrars in training. Female participants accounted for 67.5% of healthcare professionals and male participants 32.5%. Ancestry differed, with 49.8% African, 29.9% Indian, 9.6% mixed race, 9.5% Caucasian and 0.9% not associating with a defined group. Single or divorced healthcare professionals made up of 52.4% of the study population, and 47.6% were married healthcare professionals.

### Prevalence of adverse events and psychological distress

Sixty-nine (29.9%) participants reported being involved in three or more adverse obstetric events, with the majority of these participants being midwives. Most healthcare professionals reported being involved in events that resulted in morbidity or mortality (66.2%), and 19.0% of the health professionals reported being directly involved in adverse obstetric events, regardless of degree of harm. A total of 59.7% (95% confidence interval [CI]: 53.09–66.06) participants reported psychological distress. Ninety-three (40.3%) of respondents had an IES-R score ranging from 0 to 23, suggestive of no psychological distress, 29.4% (68) of respondents had IES-R scores ranging between 24 and 32, suggestive of PTSD as a clinical concern, 9.1% (21) of respondents had IES-R scores ranging between 33 and 36, suggestive of a probable diagnosis of PTSD and 21.2% (49) of respondents had IES-R scores ranging between 37 and 88, suggestive of extreme psychological distress.

### Prevalence and correlates of support-seeking behaviour

Of the 231 healthcare professionals, 65 (28.1%) sought support following an adverse obstetric event. While there was no significant correlation between age and support seeking (*p* = 0.74), the majority of those who sought support were between the ages of 30 and 39 (31.3%). A greater proportion of female healthcare professionals sought support and solicited support more frequently than male healthcare professionals, albeit non-significantly (*p* = 0.11; *p* = 0.56). Although medical officers reported seeking support more frequently than their colleagues, no significant association was noted between professional qualification and the frequency of support seeking (*p* = 0.9). Despite several healthcare professionals reporting support to be beneficial (69.2%), there were no significant correlations with age (*p* = 0.79), gender (*p* = 0.57) or professional qualification (*p* = 0.47). Knowledge of organisational support protocols in response to adverse events was reported by only 31.2% of healthcare professionals.

Healthcare professionals with greater psychological distress solicited support (*p* = 0.001). There were no associations between frequency of support and severity of psychological distress (*p* = 0.18). Healthcare professionals reporting beneficial support displayed lower psychological distress (*p* = 0.049). Those who solicited support displayed higher levels of shame (*p* = 0.046). No associations were found between support frequency (*p* = 0.31), and effectiveness of support (*p* = 0.41) and severity of feelings of shame.

## Discussion

### Key finding

Less than one-third of healthcare professionals solicited support. Midwives made up a substantial proportion of the healthcare professionals who solicited support. Healthcare professionals between the ages of 22 and 29 were the least likely to solicit support. More female participants sought support and solicited support more frequently than their male counterparts. Medical officers tended to solicit support more frequently than the other categories of healthcare professionals. Most healthcare professionals stated that soliciting support was beneficial, with more male participants reporting beneficial effects of support than female participants. The provision of organisational support for healthcare professionals involved in adverse events was low. These organisational support protocols included debriefing, referral to a psychologist and specialised support systems. Healthcare professionals with higher psychological distress were more likely to solicit support. Beneficial support lowered psychological distress in healthcare professionals. Healthcare professionals who solicited support displayed higher degrees of shame.

### Discussion of key findings

Low rate of support-seeking behaviour may be attributed to the stigma associated with mental healthcare^10^ and the fear that information may be utilised as evidence in any malpractice litigation.^11^ A substantial proportion of healthcare professionals who solicited support were midwives and may be explained as the professionals in this category were female (97.0% vs. 3.0%). Studies have found that female participants may be more likely to solicit support because of their general inclination towards psychological openness.^12^ This finding could also elucidate our finding of more female participants soliciting support than male participants, and why female participants solicited support more regularly than their male colleagues. Young healthcare professionals were less likely to solicit support after an adverse event. Similar results were found in another previous study on mental health-seeking behaviour which reported that senior individuals were more likely to seek help.^12^ In contrast, a Finnish study on general healthcare practitioners found that senior professionals develop better coping mechanisms in response to adverse events, thus making them less prone to soliciting external sources of support.^13^ The observation of gender disparity in our study where male participants reported beneficial support cannot be confirmed because of the non-availability of data from other studies; nonetheless, this discrepancy may be attributed to gender bias selection. Our finding of several healthcare professionals reporting soliciting support as beneficial is not novel and has been previously noted in studies that found support to be crucial in alleviating the negative impact of adverse events on healthcare professionals.^14^ Among the professional categories, medical officers reported soliciting support more frequently. As this study is the first study to examine healthcare professionals’ support-seeking behaviour in response to adverse obstetric events in the SA setting, our finding of the preferential support-seeking behaviour by medical officers is predictable given that competent professionals make mistakes, acknowledge them and attempt to reason the adverse event by seeking support.^13^

We report a lack of organisational support. Despite healthcare organisations being encouraged to provide support to healthcare professionals involved in adverse events, they remain largely limited or non-existent. This lack of support is highlighted in our study, which found that only 31.2% of healthcare professionals reported being aware of support provided by their organisations after adverse events. Only six healthcare professionals reported the provision of specialised support systems, 24 reported that their organisations made the provision of a psychologist and 28 reported that debriefing was the only support system provided by their organisation. Previous research shows similar results among healthcare professionals in Sweden^2^ and across primary healthcare districts and hospitals in Spain.^4^ It is important to note that our study was conducted prior to the Covid-19 outbreak. This global pandemic has since brought into light the mental health burden placed on its frontline healthcare workers. Notably the prevailing conditions during the pandemic have triggered the release of guidance and the development of tools vital to the psychological management of these professionals.^15^ In SA, the KZN Department of Health released a Covid-19 Mental Health Toolkit.^16^ We recommend that post-pandemic, this toolkit should be adapted and validated for management of second victims in healthcare organisations in SA. It may also be useful for organisations to introduce the use of support groups and implement support protocols that can be accessed anonymously to help eliminate stigma associated with asking for help.

Healthcare professionals who reported higher psychological distress solicited support. Consistent with these findings, previous studies found that support may mitigate the psychological distress caused by adverse events.^7^ Related to this finding is that psychological distress was diminished when support was perceived as beneficial. These combined findings provide circumstantial evidence that efficient support is a critical resource for all healthcare professionals. Support-seeking behaviour was also associated with increased feelings of shame for healthcare professionals which suggests that healthcare professionals may experience feelings of stigma about soliciting mental healthcare services. Other reports corroborate these findings.^11^

## Strengths and limitations

Our study allows for generalisability of organisational support because of the use of multiple public sector hospitals at central, tertiary, regional and district levels in the province. A limitation is the small sample size with the disparities between the groups and within our sample which confound analysis.

## Conclusion

To the best of our knowledge, this is the first study to examine the support offered to healthcare professionals involved in adverse obstetric events in public sector in KZN, SA. Support for healthcare professionals help alleviate second-victim responses in adverse obstetric events is necessary; however, stigma and shame emanate from soliciting support services. Considering the limited or non-existent organisational support provided by public healthcare sector in SA, we recommend the development of tools and programmes to assist second victims of adverse outcomes or at least a modification of the Covid-19 Mental Health Toolkit for broader usage. These programmes should be available to all healthcare professionals with an option to access them anonymously. In conclusion, this study albeit preliminary highlights the urgent need for support of healthcare professionals, particularly following adverse obstetric outcome.

## References

[1] Mitzman J, Jones C, McNamara S, Stobart-Gallagher M, King A. Curated collection for educators: Five key papers about second victim syndrome. Cureus. 2019;11(3):e4186. 10.7759/cureus.4186 31106086 PMC6504017

[2] Pettker CM. Systematic approaches to adverse events in obstetrics, Part II: Event analysis and response. Semin Perinatol. 2017;41(3):156–160. 10.1053/j.semperi.2017.03.004 28545653

[3] Ullström S, Sachs MA, Hansson J, et al. Suffering in silence: A qualitative study of second victims of adverse events. BMJ Qual Saf. 2013;23(4):325–331. 10.1136/bmjqs-2013-002035 PMC3963543 24239992

[4] Mira JJ, Carrillo I, Lorenzo S, et al. The aftermath of adverse events in Spanish primary care and hospital health professionals. BMC Health Serv Res. 2015;15:151. 10.1186/s12913-015-0790-7 25886369 PMC4394595

[5] Van Gerven E, Seys D, Panella M, et al. Involvement of health-care professionals in an adverse event: The role of management in supporting their workforce. Pol Arch Med Wewn. 2014;124(6):313–320. 10.20452/pamw.2297 24781784

[6] Department of Health. National policy for patient safety incident reporting and learning in the public health sector of South Africa. Pretoria: Department of Health; 2016.

[7] Sachs MA, Baehrendtz S, Sellgren SF, et al. Staff who have been involved in adverse events is left without help. Systematic support from colleagues and managers is desirable, according to interview study. Lakartidningen. 2013;110:550–552. 10.1097/NHH.0000000000000328 23596846

[8] Cauldwell M, Chappell LC, Murtagh G, et al. Learning about maternal death and grief in the profession: A pilot qualitative study. Acta Obstet Gynecol Scand. 2015;94(12):1346–1353. 10.1111/aogs.12760 26332761

[9] Weiss DS, Marmar CR. The impact of event scale – Revised. In: Wilson JP, Keane TM, editors. Assessing psychological trauma and PTSD. New York, NY: The Guilford Press, 1997; p. 399–411.

[10] Cook DR. Internalized shame scale: Professional manual. Wisconsin: Channel Press; 1994.

[11] Bell SK, Moorman DW, Delbanco T. Improving the patient, family, and clinician experience after harmful events: The ‘when things go wrong’ curriculum. Acad Med. 2010;85(6):1010–1017. 10.1097/acm.0b013e3181dbedd7 20505403

[12] DeWit ME, Marks CM, Natterman JP, Wu AW. Supporting second victims of patient safety events: Shouldn’t these communications be covered by legal privilege? J Law Med Ethics. 2013;41(4):852–858. 10.1111/jlme.12095 24446943

[13] Mackenzie CS, Gekoski WL, Knox VJ. Age, gender, and the underutilization of mental health services: The influence of help-seeking attitudes. Aging Ment Health. 2006;10(6):574–582. 10.1080/13607860600641200 17050086

[14] Nevalainen M, Kuikka L, Pitkala K. Medical errors, and uncertainty in primary healthcare: A comparative study of coping strategies among young and experienced GPs. Scand J Prim Health Care. 2014;32(2):84–89. 10.3109/02813432.2014.929820 24914458 PMC4075022

[15] Tomlin J, Dalgleish-Warburton B, Lamph G. Psychosocial support for healthcare workers during the COVID-19 pandemic. Front Psychol. 2020;11:1960. 10.3389/fpsyg.2020.01960 32849149 PMC7431467

[16] KwaZulu-Natal Department of Health. COVID-19 mental health toolkit. 2020 [cited 2020 Nov 24]. Available from: http://www.kznhealth.gov.za/mental/covid19.htm

